# Characterization and Evaluation of Alpha‐Synuclein Aggregates: Unveiling Their Potential as a Biomarker for Parkinson's Disease (PD)

**DOI:** 10.1002/alz.086109

**Published:** 2025-01-09

**Authors:** Cecile Debarnot, Malleswari Challagundla, Christoph Kleinert, Michael Schulz, Claudia Henn, Nils Boehm, Mario Richter

**Affiliations:** ^1^ University of Applied Sciences Mannheim, Mannheim, Baden‐Württemberg Germany; ^2^ AbbVie Deutschland GmbH & Co. KG, Ludwigshafen, Rheinland Pfalz Germany

## Abstract

**Background:**

Parkinson's disease (PD) is a debilitating condition that affects millions of people worldwide, yet there are currently no reliable biomarkers for its diagnosis. Alpha‐synuclein aggregation is a well‐known hallmark of PD pathology, but the behavior and kinetics of these aggregates are poorly understood. To address this gap in knowledge, this study utilized several approaches to evaluate the potential of alpha‐synuclein aggregates as potential biomarker for PD.

**Method:**

Firstly, the aggregation behavior of alpha‐synuclein (aSyn) oligomers and fibrils was evaluated using a Real‐time quaking induced conversion (RT‐QuIC) assay. This assay differentiated PD from healthy samples in human skin, brain homogenates, and cerebrospinal fluid (CSF) samples. Secondly, a symmetric ECL assay was used to quantify alpha‐synuclein oligomers and fibrils. Finally, direct stochastic optical reconstruction microscopy (dSTORM) was utilized to visualize the size and shape of recombinant alpha‐synuclein fibrils and RT‐QuIC end products (fibrils) at high resolution.

**Result:**

The results demonstrated that fast‐aggregating samples had higher concentrations of aSyn oligomers in skin and other samples compared to slow‐aggregating samples. Interestingly, human skin as a biological matrix showed a better and significant differentiation of healthy and PD samples, compared to brain or CSF indicating skin as a promising specimen for detecting alpha‐synuclein aggregation. Preliminary analysis of the average length and width of alpha‐synuclein aggregates showed promise, and further experiments with a larger sample size are required to prove whether different species of alpha‐synuclein fibrils/oligomers exist in healthy and disease samples.

**Conclusion:**

These results provide a foundation for the continued investigation of alpha‐synuclein aggregation as a potential biomarker for PD.

